# A Case of Suspected Statin-Related Delayed Onset Necrotizing Myositis

**DOI:** 10.7759/cureus.22893

**Published:** 2022-03-06

**Authors:** Elias Smirlis, Jacob Obholz, Tara Eineichner, Babajide Adio

**Affiliations:** 1 Internal Medicine Residency, MercyOne North Iowa Medical Center, Mason City, USA; 2 Internal Medicine, MercyOne North Iowa Medical Center, Mason City, USA

**Keywords:** statin, inflammatory, autoimmune, myositis, necrotizing

## Abstract

Statin-induced necrotizing myositis is a rare subtype of idiopathic inflammatory myopathies due to the production of an antibody to the 200/100 kDA protein complex which was subsequently found to be directed against 3-hydroxy-3-methylglutaryl coenzyme A reductase (HMGCR). Similar to other auto-immune necrotizing myopathies, the disease is characterized by proximal muscle weakness, significant serum creatine kinase elevations, and histological evidence of necrosis of myocytes. However, there is often little to no infiltration of inflammatory cells noted on muscle biopsy. As the name implies, this subtype of idiopathic inflammatory myopathy is provoked by statin use which may be a helpful finding during the history-taking process when developing a differential diagnosis. Below, we discuss a case of a 52-year-old female with delayed-onset immune-mediated necrotizing myopathy secondary to statin use.

## Introduction

Statins, or 3-hydroxy-3-methylglutaryl coenzyme A reductase (HMGCR) inhibitors, lower cholesterol through competitive inhibition of HMGCR, the rate-limiting step in cholesterol synthesis. In addition to their effectiveness at lowering low-density lipoprotein (LDL) and ability to improve cardiovascular outcomes, they are generally well-tolerated, making statins some of the most commonly prescribed medications [1,2]. Side effects commonly include skeletal muscle complaints ranging from myalgia to life-threatening rhabdomyolysis in extreme cases. Statin-induced myopathy is typically self-limiting and resolution of symptoms can be expected over weeks to months following cessation of statin therapy [3].

The idiopathic inflammatory myopathies include polymyositis, dermatomyositis, immune-mediated necrotizing myopathy, sporadic inclusion body myositis, and nonspecific myositis based on clinicopathological manifestations. Statin-induced necrotizing myositis is a rare subtype of idiopathic inflammatory myositis with an estimated prevalence of 2-3 in 100,000 [4]. Typical disease manifestations include proximal muscle weakness, significant serum creatine kinase elevations, and histological evidence of myocyte necrosis with little to no inflammatory infiltrate, a feature which distinguishes this disease from polymyositis or dermatomyositis [5]. Several studies have demonstrated an underlying association with auto-antibodies directed against HMGCR. In 2010, a review of a cohort of patients with clinical and/or histological evidence consistent with necrotizing myopathy identified a 200/100 kDa protein complex in 62% (16/26) of study subjects [6]. These patients did not have serologic evidence of other auto-antibodies and 63% were found to have statin exposure prior to the onset of symptom [6]. In a subsequent study of patients with necrotizing myopathy and positive auto-antibodies against the 200/100 kDa protein complex, the 100 kDa protein was identified as HMGCR [3]. After identifying the auto-antigen as HMGCR, the sera of 750 patients with evidence of myopathy were screened for anti-HMGCR auto-antibodies; 6% (45/750) of these patients were anti-HMGCR positive [3]. The anti-HMGCR positive patients had symptoms consistent with necrotizing myositis and of those greater than 50 years old, 92% had previously been exposed to statins [7].

While most statin-induced myopathies resolve after discontinuation of statin therapy, statin-induced necrotizing myositis persists or even progresses after statin withdrawal [2].

## Case presentation

A 51-year-old female with a medical history of ischemic cardiomyopathy, hyperlipidemia, hypertension, and hepatitis secondary to statin presented to the emergency department for abnormal blood work discovered by her primary care provider (PCP). The patient initially complained to her PCP about worsening muscle weakness for three weeks prior to hospital admission which prompted lab work to be performed. Initial lab work demonstrated a creatinine kinase level of 11,492 U/L, a myoglobin level of 3,662 ng/mL, an alanine aminotransferase (ALT) level of 259 IU/L, and an aspartate aminotransferase (AST) level of 249 U/L. Of note, the patient’s statin therapy had been discontinued approximately one year prior to the onset of the muscle weakness due to elevated liver enzymes including an AST level of 165 U/L and an ALT level of 321 IU/L which did resolve after a few months. At that time, the patient did not admit to experiencing any symptoms of muscle weakness. Serum studies for hepatitis B and C were also performed which were unremarkable. Creatinine kinase (CK) and aldolase levels were not performed when the patient initially had elevated liver enzymes.

Upon examination, the patient was noted to have 3/5 strength with hip flexion bilaterally and 4/5 strength with knee flexion and extension bilaterally. A 4/5 strength was noted with shoulder abduction and triceps extension bilaterally. The remainder of the patient neuromuscular exam was unremarkable.

**Figure 1:**
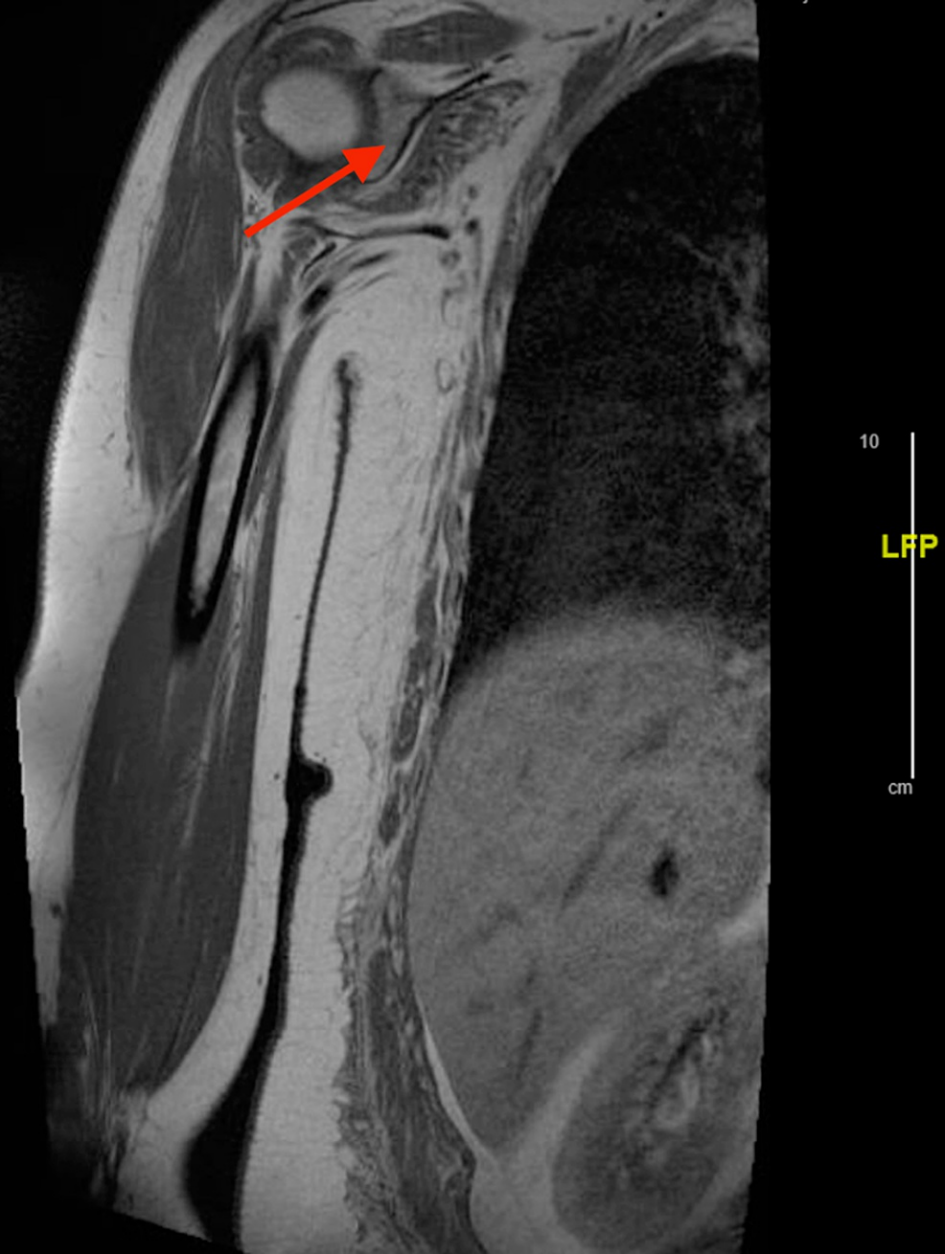
Coronal MRI of right shoulder Arrow demonstrates muscular edema of the shoulder girdle.

**Figure 2:**
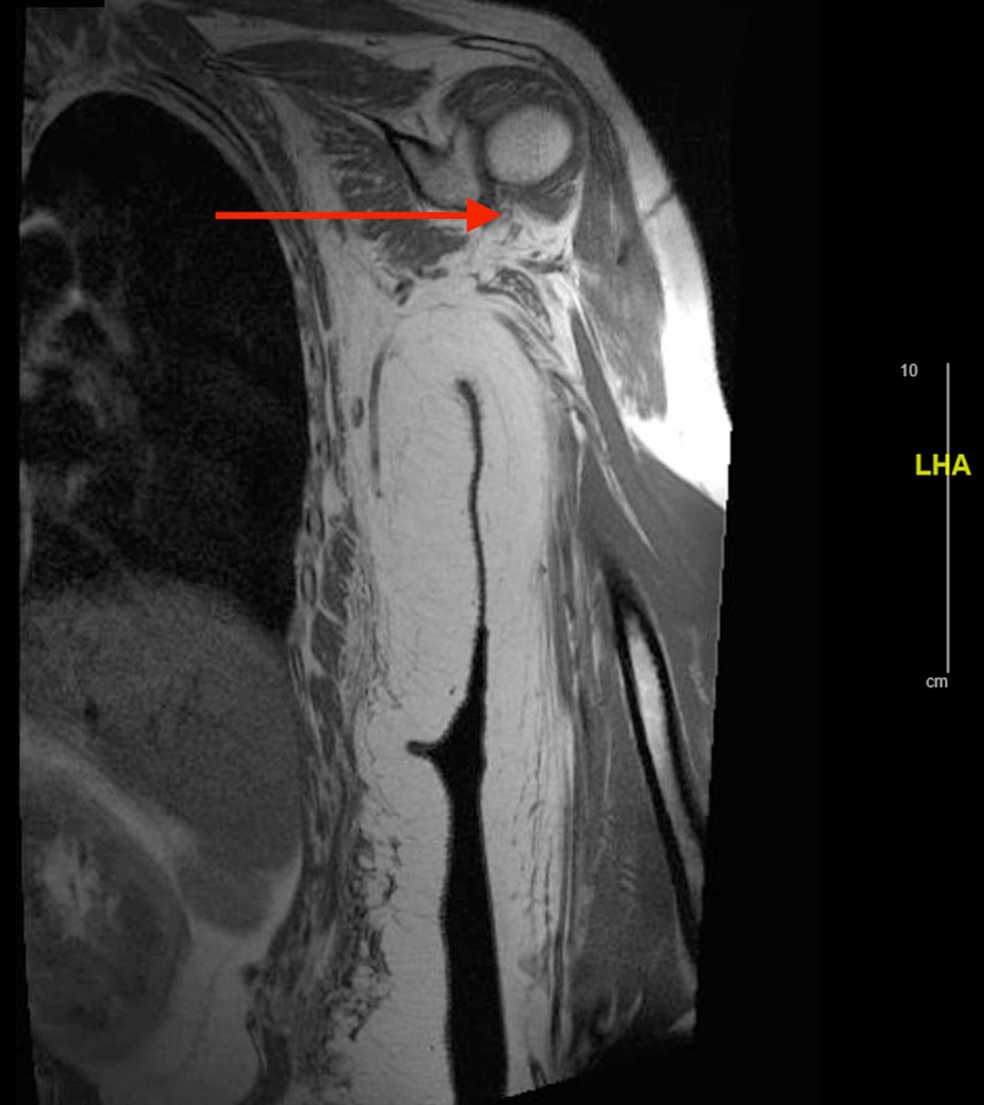
Coronal MRI of left shoulder Arrow demonstrates muscular edema of the shoulder girdle.

**Figure 3:**
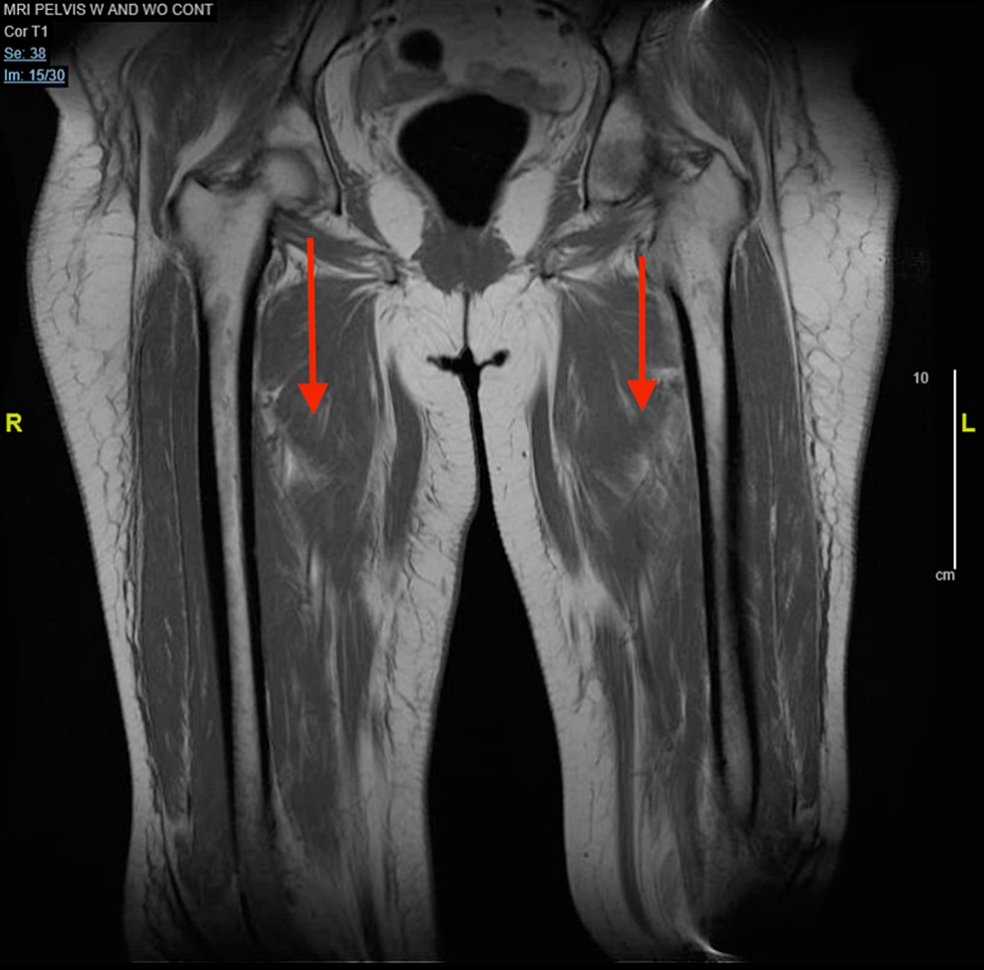
Coronal MRI of pelvis Arrows demonstrate muscular edema in the thighs bilaterally.

**Figure 4:**
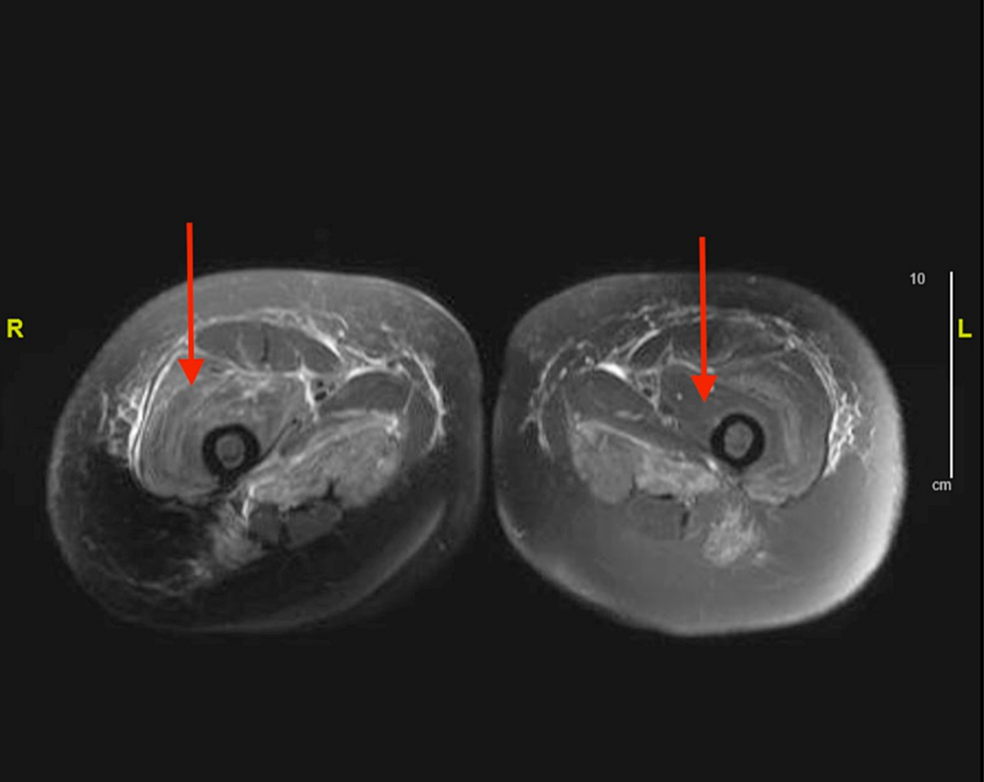
Axial MRI of pelvis Arrows demonstrate muscular edema of the thighs bilaterally.

Intravenous fluids were started for rhabdomyolysis as the patient was experiencing severe muscular weakness and her CK level was 11,908 IU/L. A workup for myositis was then initiated. Magnetic resonance imaging (MRI) of the chest and pelvis was performed in order to evaluate whether the patient had significant inflammation in these areas as the patient was experiencing the most severe symptoms in these areas. The MRI of the chest demonstrated symmetric bilateral shoulder girdle muscular edema and contrast enhancement. There was also noted perimuscular edema symmetrically within the bilateral subscapularis, teres major, supraspinatus, and latissimus dorsi muscles (Figures 1, 2). MRI of the pelvis was remarkable for bilateral muscle edema in all thigh compartments (Figures 3, 4). At this time, the rheumatology team was consulted for further evaluation. After evaluation, the rheumatology team recommended antibody testing for various autoimmune conditions and a muscle biopsy of the left quadricep muscle followed by initiation of steroid therapy. The serum autoimmune panel is shown in Table 1.

**Table 1:** Serum Autoimmune Panel Initial serum autoimmune panel ordered for evaluation and workup of patient's myositis. All results returned negative.

Serum Autoimmune Panel	
ANA	Negative
DNA double-stranded antibody	Negative
Anti-Mi-2 antibody	Negative
Anti-PL-7 antibody	Negative
Anti-EJ antibody	Negative
Anti-OJ antibody	Negative
Anti-SRP antibody	Negative
Anti-TIF-1gamma antibody	Negative
Anti-MDA-5 antibody	Negative
Anti-NXP-2 antibody	Negative
Anti-PM/Scl-100 antibody	Negative

The patient was transferred to a tertiary care facility for muscle biopsy of the left quadricep muscle and continued treatment. The muscle biopsy demonstrated mild, necrotizing myopathy with a weak multifocal expression of MHC class I which was positive for anti-HMGCR-associated immune-mediated necrotizing myopathy on immunofluorescence. Unfortunately, myopathological images were not able to be obtained. In addition, there was no lymphocytic inflammation, but scattered fibers that were undergoing myonecrosis or regeneration. Serum antibody testing was also positive with HMGCR antibody greater than 200 units. After the diagnosis was confirmed, the patient was started on intravenous immunoglobulin (IVIG) therapy in combination with glucocorticoids. The patient’s condition began to improve after treatment with IVIG and glucocorticoids, and maintenance treatment with azathioprine and tacrolimus was initiated. The patient responded well to immunotherapy and has made significant progress in her recovery to date. She has had significant improvement of the proximal muscle weakness as seen on follow-up physical exam demonstrating 5/5 strength in the shoulder abductors and elbow flexors/extensors. The patient also had improved strength to 4/5 in the hip flexors and 4/5 strength in the knee flexors/extensors. Repeat labs demonstrated a CK level of 68 U/L, ALT level of 21, AST level of 22 U/L.

## Discussion

Myalgias or other myopathic problems have been identified as a fairly common adverse side effect of 3-hydroxy-3-methylglutaryl-coenzyme A reductase inhibitors (statins) since their introduction in 1987. Over the years, observational trials have estimated the incidence of muscle-related events to range from around 9-20% which ends up affecting a significantly large population of patients given the fact that statins are one of the most widely prescribed medications in the world [1]. The degree of muscle involvement can vary heavily anywhere from benign nonspecific myalgias to fulminant rhabdomyolysis. Statin-induced necrotizing myositis was first recognized in 2010 after the discovery of the antibody to the 200/100 kDA protein complex (anti-HMGCR) which, unsurprisingly, was most common in patients being managed with statin therapy [1].

With an incidence of approximately 1 out of 10,000, statins rarely lead to serious muscle damage classified as weakness and elevated levels of creatinine kinase [7]. However, patients more often do demonstrate improvement when the offending agent is removed. Rarer still is a case of statin-induced necrotizing myositis with an estimated incidence of 2-3 per 100,000 patients treated with a statin medication [7]. Even with the removal of the statin, patients with this unique autoimmune component continue to demonstrate muscle weakness and elevated levels of creatinine kinase. Often, these patients require some level of immunosuppression to have any aspect of improvement. Patients may be on statin therapy for years prior to the development of autoantibodies leading to the necrosis of myocytes [7]. The autoimmune disease is more common in females greater than the age of 50 [8]. Patients younger than 50 often represent a subset where not only is the disease more severe and less responsive to therapy, but they are more likely to not have any exposure to a statin medication [9]. In these cases, it is postulated that exposure to statin-like compounds in some foods such as oyster mushrooms or red yeast rice may be the exacerbating factor to develop the antibody to HMGCR [9]. However, more studies need to be performed to directly link the two given the relatively low number of cases which have been reported.

Given the relative novelty and coinciding rarity of the disease, making the diagnosis can be challenging. As with all disease states, the diagnostic process should begin with taking a detailed history and physical exam. Onset of symptoms may be variable, but patients will almost always present with some degree of myalgia and proximal muscle weakness depending on the progression of the disease. Creatine kinase is uniformly elevated with a mean around 10,000 IU and is at least above 2000 IU in approximately 90% of cases [3]. Muscle edema is a common finding on MRI evaluations along with muscle atrophy and fatty replacement. Although not necessary for the diagnosis of statin-induced autoimmune necrotizing myositis, known history of statin exposure should raise concern for the disease even if statins have been discontinued for years such as in the case presented above. Detection of autoantibodies can be helpful, but many laboratories may not yet carry the proper ELISA kits or other equipment to be able to detect them. The presence of anti-HMGCR also has a low positive predictive value making it alone an insufficient diagnostic marker [1]. Muscle biopsy is often required to differentiate among similarly presenting inflammatory myopathies such as polymyositis, dermatomyositis, and inclusion body myositis. Typically, in necrotizing auto-immune myositis, muscle biopsy shows marked myonecrosis without signs of inflammation or lymphocytic infiltration [8].

Necrotizing autoimmune myositis is usually treated at tertiary centers with dedicated teams due to the rarity of the disease. The first step in therapy is the withdrawal of the offending agent if present. However, complete recovery with discontinuing the statin alone is also rare [8]. Often, patients will require some form of prolonged immunosuppression therapy; however, there are no clinical trials to date to suggest optimal management. There have been several reports of successful treatment utilizing triple therapy including high dose prednisone (usually at 1mg/kg), IVIG, and another agent such as azathioprine, methotrexate, mycophenolate mofetil, or tacrolimus [5,8]. After recovery of full strength, an attempt should be made to wean immunosuppressive therapy. Unfortunately, relapses are common especially when weaning the high-dose corticosteroid, and therapy courses can often take years [5]. Some patients are able to regain full strength on therapy despite continuously elevated CK levels. This tends to suggest that muscle regeneration and destruction occur simultaneously with muscle regeneration being the quicker process. Escalation of therapy during this process remains controversial [7]. Although muscle destruction and patient symptoms may be quite severe, with early recognition and treatment, patient outcomes are usually good with marked improvement in symptomatology.

## Conclusions

This case outlines a suspected delayed onset of HMGCR antibody formation after the use of statin therapy given the patient's age of onset, lack of symptoms previous to statin therapy, and lack of family history of any autoimmune condition. Treatment with high-dose steroid therapy in combination with at least one immunosuppressant is the current mainstay therapy although further studies need to be performed to determine optimal treatment. Early recognition and treatment often lead to symptomatic improvement in patients.

## References

[1] Hamann PD, Cooper RG, McHugh NJ, Chinoy H (2013). Statin-induced necrotizing myositis - a discrete autoimmune entity within the "statin-induced myopathy spectrum". Autoimmun Rev.

[2] Wu Y, Lach B, Provias JP, Tarnopolsky MA, Baker SK (2014). Statin-associated autoimmune myopathies: a pathophysiologic spectrum. Can J Neurol Sci.

[3] Mammen AL, Chung T, Christopher-Stine L, Rosen P, Rosen A, Doering KR, Casciola-Rosen LA (2011). Autoantibodies against 3-hydroxy-3-methylglutaryl-coenzyme A reductase in patients with statin-associated autoimmune myopathy. Arthritis Rheum.

[4] Villa L, Lerario A, Calloni S (2018). Immune-mediated necrotizing myopathy due to statins exposure. Acta Myol.

[5] Ramanathan S, Langguth D, Hardy TA (2015). Clinical course and treatment of anti-HMGCR antibody-associated necrotizing autoimmune myopathy. Neurol Neuroimmunol Neuroinflamm.

[6] Christopher-Stine L, Casciola-Rosen LA, Hong G, Chung T, Corse AM, Mammen AL (2010). A novel autoantibody recognizing 200-kd and 100-kd proteins is associated with an immune-mediated necrotizing myopathy. Arthritis Rheum.

[7] Mammen AL (2016). Statin-associated autoimmune myopathy. N Engl J Med.

[8] Allenbach Y, Drouot L, Rigolet A (2014). Anti-HMGCR autoantibodies in European patients with autoimmune necrotizing myopathies: inconstant exposure to statin. Medicine (Baltimore).

[9] Tiniakou E, Pinal-Fernandez I, Lloyd TE (2017). More severe disease and slower recovery in younger patients with anti-3-hydroxy-3-methylglutaryl-coenzyme A reductase-associated autoimmune myopathy. Rheumatology (Oxford).

