# Correction: A Case of Suspected Statin-Related Delayed Onset Necrotizing Myositis

**DOI:** 10.7759/cureus.c62

**Published:** 2022-03-28

**Authors:** Elias Smirlis, Jacob Obholz, Tara Eineichner, Babajide Adio

**Affiliations:** 1 Internal Medicine Residency, MercyOne North Iowa Medical Center, Mason City, USA; 2 Internal Medicine, MercyOne North Iowa Medical Center, Mason City, USA

The attending physician, Babajide Adio, was wrongfully excluded from this submission by the submitting author. Dr. Adio personally made contributions to the conception, design, draft and critical revision of the work prior to submission and should have been included as an author. The submitting author deeply regrets this mistake. The article has been corrected with Dr. Adio now listed as the fourth and final author.

